# Assessment of flight fatigue using heart rate variability and machine learning approaches

**DOI:** 10.3389/fnins.2025.1621638

**Published:** 2025-07-02

**Authors:** Dalong Guo, Cong Wang, Yufei Qin, Lamei Shang, Aijing Gao, Baosen Tan, Yubin Zhou, Guangyun Wang

**Affiliations:** Air Force Medical Center, Air Force Medical University, Beijing, China

**Keywords:** flight fatigue, machine learning, heart rate variability, light gradient-boosting machine, electrocardiogram

## Abstract

The accurate identification of flight fatigue is crucial for managing pilot training intensity and preventing aviation accidents. However, as a subjective perception, flight fatigue is often difficult to evaluate objectively. Heart rate variability (HRV), derived from electrocardiogram signals and regulated by the autonomic nervous system, is recognized as an effective biomarker for assessing fatigue status. This study proposes a novel HRV-based method for the automatic and objective classification of flight fatigue. This study involved an experimental investigation conducted with a cohort of 90 pilots. First, we conducted statistical analyses to investigate whether HRV features and respiratory rate indicators significantly differed across various fatigue levels. A subset of HRV features and the respiratory metric were used as input variables for four machine learning algorithms: decision tree, support vector machine, K-nearest neighbor, and light gradient-boosting machine (LightGBM). These models were applied to perform a three-level classification of flight fatigue. Finally, classification performance was evaluated using average accuracy, precision, recall, and F1 score. Among these models, LightGBM demonstrated the best performance, achieving an accuracy of 0.886 ± 0.057, precision of 0.837 ± 0.064, recall of 0.861 ± 0.086, and F1 score of 0.849 ± 0.067. These findings indicate that a LightGBM model trained on 12 selected HRV features and one respiratory indicator can accurately categorize flight fatigue into three levels. Fatigue can be detected even when mild, enabling real-time monitoring and early warning of flight fatigue. This approach holds potential for reducing fatigue-related flight accidents.

## 1 Introduction

Flight fatigue refers to the cumulative physical and mental exhaustion experienced by pilots during flight operations, which is primarily attributed to factors such as extended flight durations, circadian rhythm disruptions due to jet lag, and heightened psychological stress (Wingelaar-Jagt et al., 2021). Flight fatigue can lead to a decline in both psychological and physiological functioning in pilots, manifesting as slower reaction times, impaired judgment, and reduced motor control precision, posing serious threats to flight safety (Gaines et al., 2020; Wingelaar-Jagt et al., 2021; Jun-Ya and Rui-Shan, 2023). Historically, fatigue-related incidents have been common among military and commercial pilots (Gaines et al., 2020; Alzehairi et al., 2021; Wingelaar-Jagt et al., 2021). Such accidents are often attributed to decreased concentration and impaired performance caused by fatigue. Research has suggested that about 20% of aviation accidents are closely linked to flight fatigue (Boksem et al., 2005; Faber et al., 2012). Therefore, real-time assessment of fatigue states holds significant practical importance, as it allows for timely intervention measures to prevent major flight safety incidents (Wilson et al., 2020).

Based on the aforementioned considerations, numerous researchers have employed a variety of physiological detection techniques to assess flight fatigue. Of these, the detection of flight fatigue using an electrocardiogram (ECG) is regarded as the most promising method (Abe et al., 2014; Hu and Lodewijks, 2020; Pan et al., 2021; Li et al., 2022). Heart rate variability (HRV), obtained from processed ECG signals, has been shown to reflect autonomic nervous system activity and is widely recognized as an effective indicator for assessing drowsiness and fatigue levels in the human body (Abe et al., 2014). Furthermore, HRV monitoring serves as a non-invasive detection method that poses no risk to the pilot’s physical wellbeing and does not elicit any obvious discomfort; it can also be reliably collected through various lightweight wearable devices (Hu and Lodewijks, 2020; Chen et al., 2024). In comparison with other fatigue detection technologies such as an electroencephalogram (EEG), HRV demonstrates superior stability in-flight environments and is less susceptible to factors, including head movement, noise, light, temperature, and electromagnetic interference (Chen et al., 2024). Consequently, it provides a more precise reflection of the pilot’s actual fatigue status. In recent years, machine learning algorithms utilizing HRV have become a major focus in fatigue detection, with significant breakthroughs in the automatic analysis of mental fatigue. One study used key HRV features related to fatigue as inputs and successfully distinguished between normal and fatigued status (Wang et al., 2023). Another study used neural network algorithms to develop a driver fatigue model using the power spectral density of HRV features as input, achieving a fatigue detection accuracy of up to 90% (Patel et al., 2011). Additionally, models that employed a support vector machine (SVM) and LightGBM to classify physical fatigue also achieved promising results (Ramos et al., 2020; Cosoli et al., 2021). Research has also shown that combining ECG and respiratory signals can improve the accuracy of fatigue classification, possibly because respiratory rate (Rsp), like HRV, effectively reflects fatigue status (Chen et al., 2016; Xiang et al., 2022).

Using a range of physiological data, four supervised machine learning algorithms commonly used to classify fatigue levels are: decision tree (DT), SVM, K-nearest neighbor (KNN), and LightGBM (Kirodiwal et al., 2022; Ramkumar et al., 2023). The DT model presents its decision-making process in a tree-like structure, which facilitates an intuitive understanding of the model’s reasoning. This is particularly valuable for identifying key fatigue-related features and interpreting classification outcomes. In addition, DT models make minimal assumptions about data distribution, allowing them to be adapted to various types and distributions of fatigue-related data (Lin et al., 2010). SVM models have strong generalization ability, even when trained on limited sample sizes, which enhances their accuracy on unseen data, making it suitable for studies such as this one, where data collection can be challenging (Cervantes et al., 2020). The KNN algorithm can classify data based on the similarity of instances. It is relatively easy to implement, which allows for rapid responses to new data samples in fatigue classification tasks. It is also adaptable to various types of fatigue data and exhibits robustness against noise and outliers in the dataset (Boateng et al., 2020). LightGBM utilizes a histogram-based algorithm that enables the rapid processing of fatigue datasets, improving both training and prediction efficiency. Furthermore, LightGBM can effectively address class imbalance issues by fine-tuning its parameters, thereby enhancing the model’s classification performance (Zeng et al., 2019). From the above analysis, we conclude that all four machine learning algorithms discussed demonstrated the capability to classify fatigue states, each exhibiting distinct advantages. Consequently, this study employed these four algorithms for the automatic classification of HRV data collected during flight.

In prior studies that utilized HRV data to train machine learning models for fatigue classification, the standard 12-lead ECG acquisition equipment was predominantly employed (Rudroff, 2024). In practice, this approach is not feasible for pilots during flight as they are required to wear safety harnesses, and fighter pilots must wear anti-G suits and helmets, making the acquisition of such physiological signals impractical. Previous studies have generally classified fatigue into only two levels: fatigue and non-fatigue states (Hu and Lodewijks, 2020; Xiang et al., 2022; Chen et al., 2024). This is typically effective only for identifying severe fatigue, limiting its effectiveness for timely warning and intervention. To address these limitations, we propose an automatic classification method for flight fatigue based on wearable ECG and Rsp monitoring devices. This method involves training the four aforementioned machine learning algorithms to perform a three-level classification of flight fatigue using selected physiological features. This study aimed to evaluate the potential of wearable ECG devices in conjunction with machine learning algorithms for accurate classification and early detection of flight-related fatigue.

## 2 Materials and methods

### 2.1 Participants

We recruited 90 male Chinese volunteer pilots via volunteer programs with an average cumulative flight time of 896 ± 131 h. The mean age of the participants was 31.6 ± 6.8 years, with an average height of 173.5 ± 3.6 cm and an average weight of 73.8 ± 7.9 kg. All pilots were prohibited from consuming any medication, alcohol, coffee, tea, or other beverages that may influence their nervous systems’ excitability within 1 week prior to the flight. Additionally, all volunteers underwent rigorous physical examinations within 1 month of the experiment to ensure that they had no mental illness or sleep-related disorders. All volunteers were thoroughly informed of the potential risks prior to participation, including but not limited to drowsiness and fatigue. Written informed consent was obtained from each participant. The study protocol and measurement procedures were approved by the Ethics Committee of the Air Force Medical Center. Prior to formal testing, all participants received training to ensure proper operation of the measurement devices used in the study.

### 2.2 Data acquisition method

**FIGURE 1:**
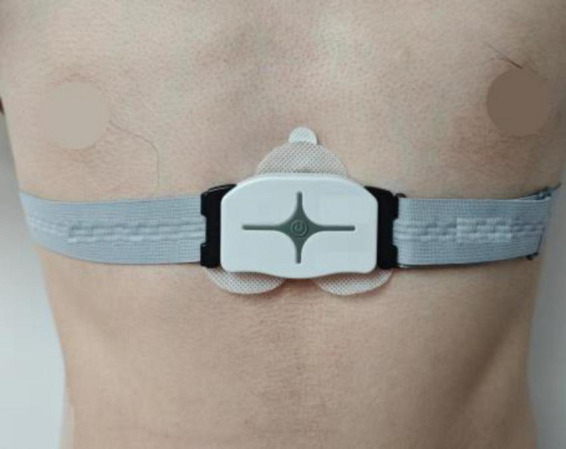
ECG chest strap placement.

As shown in Figure 1, each participant was required to wear an ECG chest strap (SensEcho, Sensecho-1A, Beijing, China) prior to the experiment. This device was specifically developed for ECG monitoring during physical activity. It uses three adhesive electrodes to record single-lead ECG signals at a sampling rate of 200 Hz. Wires embedded in the elastic chest strap also permitted respiratory signal monitoring at a sampling rate of 25 Hz. The device incorporated hardware-based noise reduction and software filtering algorithms to ensure high-quality signal acquisition. Details about the hardware design and software algorithms may be obtained from (Zhang et al., 2019).

### 2.3 Experimental procedures

Prior to data collection, all participants were introduced to the Rating of Perceived Exertion (RPE) scale and received training on its use. This scale ranges from 6 to 20, and participants were instructed to select any integer within this range that best represented their perceived levels of fatigue. Based on the RPE scores, fatigue was classified into three levels: (1) “non-fatigue,” with RPE scores of 6–10; (2) “mild fatigue,” with scores of 11–16; and (3) “severe fatigue,” with scores of 17–20 (Boksem et al., 2005; Jiang et al., 2024; Rudroff, 2024). This refined classification of fatigue levels enabled the precise evaluation and analysis of the participants’ fatigue levels, providing strong support for the accurate interpretation of the experimental data.

Participants were asked to wear the ECG monitoring device 30 min prior to flight. Baseline data were collected in a seated position, during which participants also completed the RPE scale assessment. ECG data were continuously monitored throughout the flight, and data collected within the first 5 min after flight completion were used for fatigue evaluation. Participants were again asked to complete the RPE scale immediately after data collection.

Each participant took part in at least one and up to seven data collection sessions. Flight durations varied depending on the nature of the mission, with a maximum duration of 120 min. In total, data from 392 sessions were collected. All aircraft operated by the pilots were jet fighters, and flight tasks comprised a variety of routine flight operation training exercises, including takeoff and landing procedures, instrument flight training, and aerobatic maneuver training. Table 1 describes the distribution of fatigue states across the sessions collected during the experiment. The results suggest clear individual differences in fatigue perception. For the same duration of flight, pilots reported varying levels of fatigue; however, level of fatigue may have also been influenced by the complexity of the flight tasks performed.

**TABLE 1:** Distribution of pilot flight times and fatigue states.

Flight table fatigue state	Before flight	Within 30 min	30–60 min	60–90 min	90–120 min
No. of participants in non-fatigue state	392	52	17	2	0
No. of participants in mild fatigue state	0	31	66	27	25
No. of participants in severe fatigue state	0	2	5	81	84

### 2.4 Data processing method

ECG signals were sampled at 200 Hz. To eliminate noise and motion artifacts, a low-pass filter with a cutoff frequency of 50 Hz was applied during signal preprocessing. This was conducted using the PhysioNet Cardiovascular Signal Toolbox (Clifford Lab, Emory University, Atlanta, GA, United States) in MATLAB R2018a (Vest et al., 2018). The selection of HRV features played a critical role in developing a model with high sensitivity and specificity for fatigue detection. Including irrelevant or redundant information related to fatigue can increase computational cost and the risk of overfitting during model training. Based on the preprocessed ECG and respiratory signals and previous findings (Zeng et al., 2019; Wang et al., 2023; Rudroff, 2024), 16 ECG-derived HRV features and one respiratory feature with demonstrated sensitivity to fatigue were chosen. Table 2 presents the names and descriptions of these features.

**TABLE 2:** Extracted ECG and respiratory features.

Type of feature	Feature	Description
ECG features	Mean HR	Mean heart rate
	Mean RR	Mean R–R interval (the time between successive heartbeats)
	SDNN	Standard deviation of all RR intervals
	SDSD	Standard deviation of differences between adjacent RR intervals, reflecting short-term variability
	RMSSD	Root mean square of successive RR interval differences
	pNN20	Percentage of successive RR intervals that differ by more than 20 ms
	pNN50	Percentage of successive RR intervals that differ by more than 50 ms
	LF	Absolute power of low-frequency band (0.04–0.15 Hz)
	LFn	Normalized low-frequency (LF) power (i.e., LF divided by total power)
	HF	Absolute power of high-frequency band (0.15–0.4 Hz)
	HFn	Normalized high-frequency (HF) power (HF divided by total power)
	LF/HF	Ratio of low-frequency to high-frequency power
	S	Total HRV ellipse area, proportional to SD1 and SD2
	SD1	Standard deviation of instantaneous beat-to-beat variability (short-term HRV, perpendicular to line-of-identity in Poincaré plot)
	SD2	Standard deviation of continuous long-term RR variability (along the line-of-identity in Poincaré plot)
	SD1/SD2	Ratio of short-term to long-term HRV
Respiratory indicator	MeanRsp	Mean respiratory rate

Existing research indicates that, during states of fatigue, the sympathetic nervous system (SNS) becomes suppressed, leading to slower energy metabolism, delayed responses, and reduced mental alertness. Conversely, the parasympathetic nervous system (PNS) becomes more active, inhibiting SNS activity, reducing heart rate, and promoting rest and recovery. In fatigued states, time-domain HRV features—such as mean heart rate (HR), mean R–R interval (RR), root mean square of successive differences (RMSSD), standard deviation of normal-to-normal intervals (SDNN), and the percentage of successive RR intervals that differ by more than 50 ms (pNN50)—typically show an increase. These changes reflect enhanced parasympathetic regulation, as the body attempts to conserve energy and promote recovery by reducing heart rate and increasing HRV. In terms of frequency-domain indicators, low-frequency (LF) power increases, high-frequency (HF) power decreases, and the LF/HF ratio rises. These changes suggest a relative dominance of sympathetic activity and a reduction in parasympathetic regulation, indicating that the autonomic nervous system is in a state of stress during fatigue (Ni et al., 2022).

### 2.5 Machine learning methods

The performance of the classification models was evaluated using the following metrics: accuracy, defined as the proportion of correctly predicted samples out of the total number of samples; precision, the proportion of true-positive predictions among all samples predicted as positive; recall, the proportion of true-positive predictions among all actual positive samples; and F1 score, the harmonic mean of precision and recall (Dauner et al., 2023). The mathematical formulas for these four evaluation metrics are defined as follows (Dauner et al., 2023; Zahid et al., 2023):


(1) $$A\,c\,c\,u\,r\,a\,c\,y=\frac{T\,P+T\,N}{T\,P+F\,P+T\,N+F\,N},$$

(2) $$P\,r\,e\,c\,i\,s\,i\,o\,n=\frac{T\,P}{T\,P+F\,P},$$

(3) $$R\,e\,c\,a\,l\,l=\frac{T\,P}{T\,P+F\,N},$$

(4) $$F\,1=\frac{2\,P\,r\,e\,c\,i\,s\,i\,o\,n\,R\,e\,c\,a\,l\,l}{P\,r\,e\,c\,i\,s\,i\,o\,n+R\,e\,c\,a\,l\,l}.$$

In this context, true positive (TP) refers to the number of samples correctly classified into a given category. False positive (FP) represents the number of samples incorrectly classified into a category to which they do not belong. True negative (TN) denotes the number of correctly classified samples that belong to another category, while false negative (FN) refers to the number of samples that belong to a given category but are misclassified into another category. The average values of these metrics were calculated to obtain the final performance evaluation of the model.

Tenfold cross-validation was used to assess model performance. To prevent data from the same participant being used in both the training and testing sets, each iteration used data from 81 participants for training and data from the remaining nine participants for testing. Average performance across the 10 iterations was reported as the final result. Additionally, to ensure that the test samples were representative, each testing set was required to include at least nine samples for each of the four flight duration categories (less than 30 min, 30–60 min, 60–90 min, and 90–120 min). In the above-mentioned method, all four models employed the same proportion of training and test sets. This was done to eliminate performance evaluation differences caused by different data division ratios, evaluate the performance stability of the models more accurately, and reduce the errors caused by the randomness of data division. As a unified data division ratio can simulate the same data environment, this enabled a better comparison of the model’s performance during practical use.

Using SPSS^®^ Statistics version 26.0 (IBM Corp, Armonk, NY, United States), we performed statistical analyses to examine the differences in ECG and respiratory features across different states of fatigue. First, normality tests were performed to determine whether the data conformed to normal distribution. For features that met the assumption of normality, one-way repeated-measures analysis of variance (ANOVA) was used to assess differences among flight fatigue states. For features that did not meet the assumption of normality, the Kruskal–Wallis H-test was used to examine differences across multiple groups. A *p*-value of < 0.05 was considered statistically significant.

## 3 Results

### 3.1 Feature selection for model input

As shown in Tables 3, 4, homogeneity of variance tests was conducted for all ECG indicators. Mean HR, mean RR, SDNN, RMSSD, and SD2 met the assumption of homogeneity and were analyzed using ANOVA. In contrast, SDSD, pNN50, pNN20, LF, HF, LF/HF, LFn, HFn, SD1, SD1/SD2, and S did not meet the assumption and were analyzed using the Kruskal–Wallis *H*-test. Among these features, mean HR, mean RR, SDNN, RMSSD, pNN50, pNN20, LF, HF, LF/HF, LFn, HFn, and SD1/SD2 showed statistically significant differences across different states of fatigue. For the respiratory indicator, mean Rsp met the homogeneity of variance assumption and was tested using ANOVA, which indicated significant differences across fatigue states.

**TABLE 3:** Results for differences in ECG and respiratory features (ANOVA test).

Feature	Unit	*F*-value	*P*-value
Mean HR	Times/min	6.262	< 0.001**
Mean RR	Ms	5.731	< 0.001**
SDNN	Ms	4.152	0.028*
RMSSD	ms	3.621	0.031*
SD2	ms	1.061	0.513
Mean Rsp	Times/min	5.268	< 0.001**

**p < 0.01, *p < 0.05.

**TABLE 4:** Results of differences in ECG features (Kruskal–Wallis *H*-test).

Feature	Unit	*H*-value	*P*-value
SDSD	ms	5.182	0.075
pNN50	%	8.752	0.007**
pNN20	%	9.281	0.002**
LF	ms^2^	6.723	0.047*
HF	ms^2^	6.983	0.042*
LF/HF	/	20.678	< 0.001**
LFn	/	19.525	< 0.001**
HFn	/	15.627	< 0.001**
SD1	ms	5.293	0.073
SD1/SD2	/	16.739	< 0.001**
S	/	4.134	0.161

**p < 0.01, *p < 0.05.

Based on the results presented above, 12 ECG features and one respiratory indicator exhibited statistically significant differences across the various flight fatigue states.

### 3.2 Training results of the flight fatigue classification model

A total of 12 ECG features and one respiratory indicator that demonstrated statistically significant differences across different fatigue states were used as input variables for the models. The ECG features included mean HR, mean RR, SDNN, RMSSD, pNN50, pNN20, LF, HF, LF/HF, LFn, HFn, and SD1/SD2. The respiratory feature was mean Rsp. The DT, SVM, KNN, and LightGBM algorithms were used to construct the classification models.

During model development, the grid search algorithm was used to optimize the hyperparameters of the classifier to systematically search for the best hyperparameter combination within the specified range to improve the performance of the model. For each parameter combination, the average accuracy for that combination was calculated using tenfold cross-validation, and the parameter combination with the highest average accuracy of the classifier was selected as the final result. For the DT model, the parameter space was defined in terms of “max_depth”: [None, 3, 6, 9] and “criterion”: [“Gini,” “entropy”]; the optimal parameter combination was a maximum tree depth of 3 and a splitting criterion of Gini index. For the SVM model, the parameter space was defined in terms of “C”: [0.1, 1, 10] and “kernel”: [“linear,” “rbf,” “poly”], with the optimal regularization parameter set to C = 1 and the kernel parameter set to “rbf.” For the KNN model, the parameter space was defined as follows: “n_neighbors”: [3,5,7] and weights”: [“uniform,” “distance”]. The optimal configuration was found with *K* = 5 and “distance” as the weighting method. For the LightGBM model, the parameter space included “num_leaves”: [10, 20, 30, 40, 50, 60] and “learning_rate”: [0.01, 0.05, 0.1, 0.2, 0.3]. The optimal parameters were a maximum of 60 leaves and a learning rate of 0.01.

Table 5 lists the average accuracy, precision, recall, and F1 score for each of the four machine learning models using the same feature set to evaluate flight fatigue. The results indicated that the LightGBM model outperformed all other models across all performance metrics, achieving an accuracy of 0.886 ± 0.057, precision of 0.837 ± 0.064, recall of 0.861 ± 0.089, and F1 score of 0.849 ± 0.067. These results demonstrate the feasibility of using HRV features and the respiratory indicator to perform a three-level classification of flight fatigue.

**TABLE 5:** Performance of four machine learning models using different feature sets.

Model	Accuracy	Precision	Recall	F1
DT	0.851 ± 0.073	0.821 ± 0.086	0.759 ± 0.087	0.783 ± 0.091
KNN	0.863 ± 0.062	0.831 ± 0.084	0.776 ± 0.069	0.789 ± 0.078
SVM	0.851 ± 0.068	0.796 ± 0.072	0.823 ± 0.079	0.824 ± 0.063
LightGBM	0.886 ± 0.057	0.837 ± 0.064	0.861 ± 0.086	0.849 ± 0.067

**FIGURE 2:**
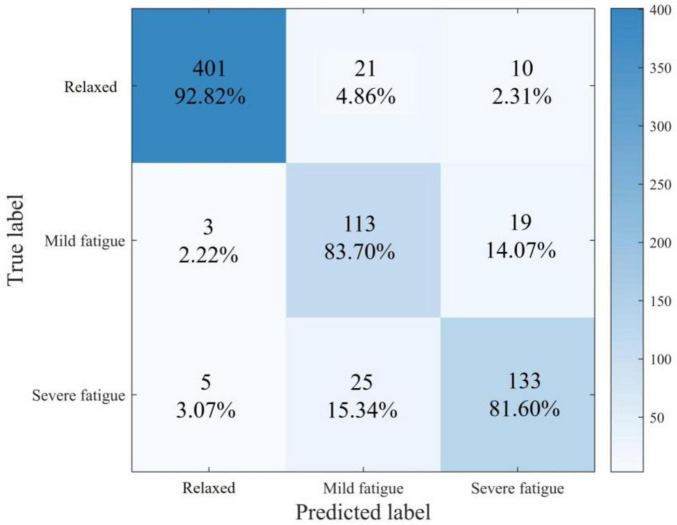
Overall confusion matrix of the LightGBM model based on tenfold cross-validation.

Figure 2 shows the overall confusion matrix of the LightGBM model in the 10-fold cross-validation process. The row labels represent the true class of each sample, while the column labels represent the predicted class. The color shading of each cell represents the proportion of samples in that row assigned to the corresponding prediction category.

## 4 Discussion

This study employed several HRV features—mean HR, mean RR, SDNN, RMSSD, pNN50, pNN20, LF, HF, LF/HF, LFn, HFn, and SD1/SD2—and a respiratory indicator, MeanRsp to assess flight fatigue. While these features have been widely used in prior studies to assess physiological fatigue induced by exercise or mental fatigue resulting from prolonged cognitive tasks (Rudroff, 2024), we demonstrate their applicability in evaluating flight-related fatigue. This is plausible given the nature of flight fatigue, which is a complex state resulting from high workloads, demanding flight tasks, and circadian rhythm disruptions – factors that contribute to both the physiological and psychological components of fatigue (Sun et al., 2024).

As recorded in Table 5, LightGBM outperformed other models across all performance metrics. This superiority is likely attributable to its gradient-boosting DT framework, which captures nonlinear relationships and allows for more precise data partitioning and fitting, thereby enhancing model accuracy. In many real-world classification tasks, prior studies have also demonstrated that LightGBM achieves higher classification accuracy than traditional models, such as logistic regression and SVM, in various tasks (Wei et al., 2023). Moreover, previous studies have shown that LightGBM is effective for classifying muscle and mental fatigue caused by prolonged physical activity or driving (Zeng et al., 2019).

A previous study (Ni et al., 2022) combined LightGBM with a 12-lead ECG device for fatigue classification, achieving a maximum accuracy of 0.855 and an F1 score of 0.801. These results are comparable to those of the current study despite using a single-lead ECG device. This can primarily be attributed to comprehensive utilization of ECG and respiratory indicators, as well as the more refined optimization of these indicators. This supports the feasibility of using wearable ECG devices for accurate flight fatigue classification.

Based on the results exhibited in Figure 2, the model performed best when predicting the “non-fatigue” state, achieving 92.82% accuracy. It performed the worst in predicting “severe fatigue” states, with only 81.60% accuracy. Examination of the confusion matrix reveals that this is mainly attributable to the model’s reduced sensitivity in distinguishing between “mild fatigue” and “severe fatigue” samples, misclassifying 15.34% of “severe fatigue” samples as “mild fatigue” and 14.07% of “mild fatigue” samples as “severe fatigue.” A key factor contributing to this limitation is class imbalance within the dataset. The dataset contained a higher number of “non-fatigue” samples than “mild fatigue” and “severe fatigue” samples. The limited number of fatigue samples restricted the classifier’s ability to learn robust features for those states, reducing its generalization performance when predicting fatigue states.

In terms of detecting flight fatigue, the LightGBM model in this study demonstrated an accuracy of 0.886 ± 0.057, precision of 0.837 ± 0.064, recall rate of 0.861 ± 0.086, and F1 score of 0.849 ± 0.067. These results indicate that a model trained using HRV and respiratory rate exhibits high overall performance in the three-class classification task of flight fatigue and is capable of accurately distinguishing samples across different fatigue levels. Previous studies focused on flight fatigue detection based on EEG have achieved similar high accuracy rates, with some reporting accuracies ranging from 0.8 to 0.9 (Lee et al., 2020; Wu et al., 2021; Alreshidi et al., 2023; Lee et al., 2023; Taheri Gorji et al., 2023). However, the acquisition of EEG signals typically necessitates complex equipment and specialized electrode placement procedures, and is susceptible to external electromagnetic interference and other factors, leading to relatively inferior signal stability and repeatability. By contrast, the chest strap method employed in this study for detecting HRV and respiratory rate enables more stable data collection, is less vulnerable to external interference, and achieves comparable model performance to the EEG-based approach, thereby demonstrating certain advantages.

Several other studies have utilized eye movement indicators, such as blink frequency and eyelid closure time, for flight fatigue detection, achieving accuracy rates that range from 0.7 to 0.8 (Borghini et al., 2014; Qin et al., 2021; Xue et al., 2022). However, eye-tracking devices face significant challenges related to installation and operational usability in real-world flight environments. Additionally, pilots may wear goggles or helmets during flights, which could interfere with the acquisition of eye movement indicators. The chest strap detection method employed in this study was unaffected by visual assistive devices, thereby enhancing the reliability of data collection and surpassing some studies based on eye movement indicators in terms of detection accuracy.

Finally, another category of research focused on detecting fatigue by analyzing pilots’ operational actions, reaction times, and other behavioral characteristics, achieving accuracy rates that typically range from 0.6 to 0.75 (Zhou et al., 2022; Wang et al., 2023; Ghaderi and Saghafi, 2024). However, behavioral and operational indicators are susceptible to various factors, such as flight mission types and flight environments, and thus exhibit relatively low specificity. By contrast, the physiological indicators (HRV and MeanRsp) examined in this study can reflect the status of the pilot’s autonomic nervous system and fatigue level more directly, thereby demonstrating an advantage in terms of detection accuracy.

This study employed the chest strap method for measuring HRV and MeanRsp, which offers high measurement convenience. The chest strap is a lightweight and easy-to-wear device that does not interfere with the pilot’s operation during flight nor impose any additional burden. Its installation and use are straightforward, requiring only that it be wrapped around the chest and secured, without the need for complex settings or calibration processes. In addition, chest strap devices typically possess wireless data transmission capabilities, enabling real-time data transfer to the monitoring system and thereby facilitating continuous flight monitoring. Conversely, EEG detection necessitates the placement of multiple electrodes on the scalp, along with the use of auxiliary materials such as conductive paste to ensure optimal signal quality. This procedure is not only cumbersome and time-consuming, but may also cause discomfort for the pilot (Lee et al., 2020; Lee et al., 2023). In actual flight scenarios, particularly in emergency flight missions or during long-duration flights, pilots may be unwilling to allocate a significant amount of time for the installation and debugging of EEG equipment (Alreshidi et al., 2023; Taheri Gorji et al., 2023).

Eye-tracking devices generally require precise installation, either on the pilot’s head or within the cockpit, along with a rigorous calibration process to ensure the accurate collection of eye movement data. During flight operations, the pilot’s head movements and changes in the cockpit environment, such as fluctuations in lighting conditions, may interfere with the performance of the eye-tracking device, potentially resulting in data loss or an increase in measurement errors (Qin et al., 2021; Xue et al., 2022). However, the chest strap detection method remains unaffected by factors such as head movement and lighting conditions, thus ensuring a more stable and convenient measurement process. Additionally, there have been studies utilizing blood oxygen sensors placed on the fingers or earlobes to detect indicators such as pulse oxygen saturation for assessing flight fatigue (Sammito et al., 2022; Ibrahim et al., 2024). Although these sensors provide a certain level of convenience, they are typically limited to detecting a single physiological indicator, whereas the chest strap employed in this study is capable of simultaneously measuring two critical physiological indicators. This provides more comprehensive fatigue-related information, thereby facilitating a more accurate assessment of pilots’ fatigue states.

Furthermore, the model developed in this study was both trained and validated under actual in-flight scenarios, yielding results with high reliability. Given that flight fatigue is influenced by a multitude of factors, including flight altitude, pilot posture, and task load, the actual flight environment can effectively capture the true impact of these variables on pilots’ fatigue states (Jun-Ya and Rui-Shan, 2023). Many prior studies on flight fatigue have been conducted using ground-based simulators that primarily collected physiological data under controlled laboratory conditions for fatigue analysis (Alzehairi et al., 2021; Chen et al., 2024). While they may enable in-depth exploration of the relationship between physiological indicators and fatigue, the absence of actual flight-related background and environmental factors (such as turbulence and air current variations) may limit the reliability and applicability of their findings in real-world flight fatigue monitoring. This study performed measurements and model training during actual in-flight operations, thereby avoiding the discrepancies that may arise between simulator-based environments and real flight conditions. Consequently, the findings gained greater reliability and practical applicability.

The automatic three-classification method for flight fatigue developed in this study holds broad application potential. Its high detection accuracy and convenient measurement process enable seamless integration into existing aviation flight monitoring systems, providing real-time fatigue state monitoring and early warning capabilities for pilots. By promptly identifying the fatigue state of pilots, particularly during the early stages of mild fatigue, appropriate measures (such as adjusting flight task schedules or reminding pilots to rest) can be implemented to prevent fatigue from escalating, thereby significantly reducing the risk of fatigue-induced flight accidents. Some prior studies only achieved binary classification of fatigue versus non-fatigue states, which, while capable of identifying fatigue to a certain extent, may have limitations in effectively managing flight fatigue (Ramos et al., 2020). As pilot fatigue is a gradual process, the binary classification method fails to precisely quantify the severity of fatigue and struggles to detect fatigue in its early stages, thereby hindering timely implementation of targeted measures.

Moreover, some studies rely solely on a single fatigue indicator for detection (Lee et al., 2023; Taheri Gorji et al., 2023). Due to the lack of integration of multiple fatigue-related factors, these methods may yield incomplete or inaccurate results. In real-world flight scenarios, fatigue arises from the synergistic effects of various physiological and psychological factors. Consequently, detection methods that incorporate multiple indicators can provide a more accurate assessment of the fatigue state. This study integrated two critical physiological indicators and performed a comprehensive analysis using the LightGBM model. This approach enables more effective detection of flight fatigue and better aligns with the requirements of real-world flight fatigue monitoring, demonstrating broader application potential.

This study utilized a chest strap to measure pilots’ HRV and MeanRsp during actual flight operations, and applied the LightGBM machine learning model to achieve automated three-class classification of flight fatigue. This approach demonstrates certain advantages in terms of detection accuracy, measurement convenience, result reliability, and application potential. Compared with prior studies that relied on EEG, eye movement indicators, behavioral and operational metrics, as well as ground-based simulators, the method employed in this study is better suited for real flight environments and can provide more precise, reliable, and timely results for flight fatigue monitoring. Furthermore, while this study only utilized MeanRsp among the respiratory indicators, the chest strap employed can also measure inspiratory time, expiratory time, the ratio of inspiratory to expiratory time, and the high-frequency and low-frequency components of the respiratory wave. In future research, these additional indicators could be incorporated comprehensively to train machine learning models, potentially leading to even more accurate models.

This study has several limitations. Owing to the unique nature of the profession, the number of participants was relatively small, which might have affected the model’s stability and generalizability. In the future, it is advisable to monitor the HRV and respiratory indicators of a larger number of pilots, particularly those involved in long-duration flights and high-complexity missions. Additionally, we did not separately investigate physical and mental fatigue but instead adopted a general classification of flight fatigue. As a result, the HRV features used in the model might not represent the optimal input set for model training. Future research should focus on identifying the most effective HRV features specific to the characteristics of flight fatigue. In future research, it is essential to differentiate between these two distinct fatigue states and to monitor various forms of flight-related fatigue independently. Finally, this study focused exclusively on the physiological parameters of male pilots. Given that women possess distinct physiological structures and are potentially more susceptible to fatigue, future research should also consider conducting separate classification analyses of flight fatigue among female pilots.

## 5 Conclusion

This study explored the use of HRV features and respiratory indicators to automatically assess flight fatigue involving a sample of 90 pilots based on machine learning. Statistical analysis was first used to select relevant features, which included 12 HRV features—mean HR, mean RR, SDNN, RMSSD, pNN50, pNN20, LF, HF, LF/HF, LFn, HFn, and SD1/SD2—as well as one respiratory indicator, mean Rsp. These were used as inputs for four machine learning algorithms: DT, SVM, KNN, and LightGBM. Among these, LightGBM demonstrated the best performance, confirming the feasibility of using HRV and respiratory features for flight fatigue assessment.

## Data Availability

The original contributions presented in this study are included in this article/supplementary material, further inquiries can be directed to the corresponding authors.
